# Tumor necrosis factor-α–stimulated gene 6 promotes hematoma clearance after intracerebral hemorrhage in a mouse model

**DOI:** 10.4103/NRR.NRR-D-24-00968

**Published:** 2025-06-19

**Authors:** Xia Liu, Dabao Yao, Yunjie Li, Shiling Chen, Yingxin Tang, Jingyi Wang, Jingfei Yang, Jie Jing, Jiahui Wang, Ge Zhang, Luwei Nie, Yangyang Feng, Gaigai Li, Zhouping Tang

**Affiliations:** 1 Department of Neurology, Tongji Hospital, Tongji Medical College, Huazhong University of Science and Technology, Wuhan, Hubei Province, China; 2 Department of Neurology, Jingzhou Hospital, Yangtze University, Jingzhou, Hubei Province, China; 3 Hubei Key Laboratory of Neural Injury and Functional Reconstruction, Huazhong University of Science and Technology, Wuhan, Hubei Province, China; 4 Department of Nuclear Medicine, Tongji Hospital, Tongji Medical College, Huazhong University of Science and Technology, Wuhan, Hubei Province, China; 5 Department of Neurology, Qilu Hospital, Shandong University, Jinan, Shandong Province, China

**Keywords:** apoptosis, growth arrest–specific protein 6, hematoma absorption, inflammation, intracerebral hemorrhage, iron deposition, microglia/macrophages, polarization, signal transducer and activator of transcription 6, tumor necrosis factor-α–stimulated gene 6

## Abstract

The prognosis for patients who experience intracerebral hemorrhage is poor because of a lack of effective treatments. Tumor necrosis factor-α–stimulated gene 6 (TSG6) is a secreted glycoprotein that exerts anti-inflammatory effects in various inflammatory diseases. We previously showed that adipose-derived stem cells can inhibit inflammation by upregulating TSG6 secretion in an *in vitro* model of intracerebral hemorrhage. However, the direct effects of TSG6 on hematoma clearance *in vivo* remain largely unknown. The aim of this study was to determine how TSG6 affects hematoma absorption in mice subjected to intracerebral hemorrhage and to explore the potential underlying mechanisms. We first analyzed the gene profiles of patients with intracerebral hemorrhage from the GEO database and examined changes in TSG6 expression in the brain tissues of mice subjected to intracerebral hemorrhage. We found that TSG6 expression exhibited a transient increase following intracerebral hemorrhage, and that there was a negative correlation between the initial hematoma volume and TSG6 levels. Immunofluorescence analysis showed that TSG6 was primarily expressed in microglia and macrophages. Furthermore, we found that TSG6 promoted functional recovery in mice subjected to intracerebral hemorrhage by accelerating hematoma clearance, reducing the number of apoptotic cells and degenerated neurons, increasing the proportion of phagocytic microglia/macrophages, and decreasing iron deposition. Western blotting and immunofluorescence analysis indicated that TSG6 promoted M2 polarization of microglia/macrophages. *In vitro* phagocytosis experiments confirmed that TSG6 enhanced the ability of microglia to phagocytize red blood cells. Finally, we identified the signal transducer and activator of transcription 6/growth arrest–specific protein 6 signaling pathway as playing a critical role in TSG6-mediated hematoma absorption. In summary, our results demonstrate an essential role for TSG6 in promoting hematoma absorption in a mouse model of intracerebral hemorrhage. These findings suggest that TSG6 accelerates hematoma clearance and improves neurological function by promoting microglia/macrophage polarization to the M2 phenotype, activating the STAT6/GAS6 signaling pathway, and increasing phagocytic receptor expression on the surface of phagocytes, thereby enhancing their ability to phagocytize red blood cells.

## Graphical abstract

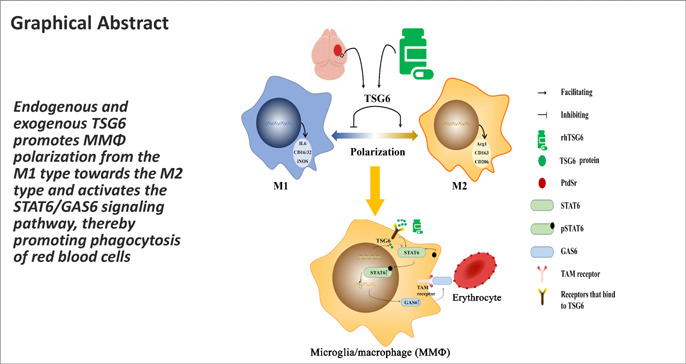

## Introduction

Intracerebral hemorrhage (ICH) is a common neurological event with high fatality and disability rates (Xue and Yong, 2020). The majority of patients who survive ICH are unable to live independently and have a higher chance of developing other nervous system disorders, such as dementia (Cordonnier et al., 2018). ICH is typically treated by surgically removing the hematoma, but the efficacy and safety of this approach are undetermined. The MISTIE Phase III clinical trial study demonstrated that removing hematomas through minimally invasive surgery and local application of the thrombolytic medication alteplase can lower patient mortality, and that removing hematomas is strongly associated with improved neurological scores (Awad et al., 2019; Hanley et al., 2019). This finding suggests that key measures for treating ICH are effectively removing the hematoma as early as possible and eliminating its toxic effects. The complex pathological process of ICH may hinder complete removal of the hematoma regardless of whether a craniotomy or a minimally invasive procedure is performed. Residual hematoma metabolism can further exacerbate brain edema and inflammatory damage, which can hinder the patient’s ability to regain neurological function (Awad et al., 2019; Jelinek and Duris, 2023). Furthermore, no medication has yet been shown to effectively lead to hematoma absorption or clearance. Hematoma clearance is mostly mediated by the endogenous hematoma clearance pathway, particularly for patients with small hematomas who do not need surgery (Awad et al., 2019). Thus, it is crucial to investigate the pathophysiological mechanisms of endogenous hematoma clearance, decrease the residual volume of large hematomas, and facilitate the absorption of small hematomas to foster functional recovery in patients with ICH.

Following activation, monocytes and microglia develop into macrophages, which can secrete various cytokines and release receptors associated with phagocytosis. During this transition, the cell morphology shifts from branching to amoeba-like (Bai et al., 2020; Tschoe et al., 2020). It is generally difficult to distinguish between activated microglia and peripheral macrophages using morphology and markers; therefore, they are collectively referred to as microglia/macrophages (MMΦ; Xue and Yong, 2020). Activated MMΦ can be divided into classically activated MMΦ (M1 type) and alternatively activated MMΦ (M2 type), depending on their functional state. M2-type MMΦ include three subtypes: M2a, M2b, and M2c. Unlike the M1 type, M2-type MMΦ mainly play an anti-inflammatory role, with M2b/M2c-type cells engaging in phagocytosis and clearing tissue debris (Lan et al., 2017; Jin et al., 2021). Microglia are mainly found in the central nervous system and are responsible for mediating phagocytosis and hematoma clearance (Jin et al., 2021). Phagocytosis is the process by which phagocytes recognize, internalize, and engulf cell debris or other particles using receptors, form phagosomes, and combine with lysosomes to digest and eliminate the ingested substances (Li et al., 2021). Phosphatidyl serine (PtdSer) is the most ubiquitous “eat me” signal protein on the surface of cells that leads to their phagocytosis. Following cell death, PtdSer on the inside of the cell membrane is exposed on the outside of the cell membrane, allowing phagocytes to recognize and distinguish dead cells from living cells (Lemke, 2019). Apoptotic cells attract circulating MMΦ, which recognize and ingest the apoptotic cells via receptors with exposed “eat me” signals (Li et al., 2021).

As an intermediary protein, growth arrest–specific protein 6 (GAS6) can link the tyrosine kinase receptors Anexelekto (AXL) and Mer receptor tyrosine kinase (MERTK) on the surface of phagocytes to PtdSer on the surface of apoptotic cells, hence promoting activation of these receptors (Rothlin et al., 2015; Fourgeaud et al., 2016). Previous research has demonstrated that AXL and MERTK play a critical role in hematoma clearance by enhancing macrophage-mediated phagocytosis of red blood cells (RBCs) following ICH (Fourgeaud et al., 2016). When the GAS6 gene is knocked out, macrophage-mediated phagocytosis of apoptotic neutrophils is significantly reduced. This reduction is due to less GAS6 protein being available to act on tumor-associated macrophage (TAM) receptors (Nepal et al., 2019). However, whether GAS6 is involved in MMΦ-mediated phagocytosis of erythrocytes after ICH remains undetermined.

In recent years, stem-cell therapy has gained increasing recognition as a novel approach to treating ICH (Monsour and Borlongan, 2023; Wang et al., 2025). Mesenchymal stem cells (MSCs) secrete glycoprotein tumor necrosis factor-α-stimulated gene 6 (TSG6), which has a molecular weight ranging from 35 to 38 kDa (Day and Milner, 2019). TSG6 is found in extracellular vesicles derived from MSCs and is crucial for stem cell–mediated immune regulation (Roura et al., 2020). Adipose-derived stem cells have been shown to prevent BV2 microglia–mediated inflammation by secreting TSG6 (Hu et al., 2018). Additionally, dynamic changes in TSG6 expression were observed in rats post–subarachnoid hemorrhage (Li et al., 2018). However, little is known about the direct pathophysiological effects of TSG6 in ICH and the underlying mechanisms. Studies have shown that TSG6 activates signal transducer and activator of transcription 6 (STAT6; Nepal et al., 2019; Liu et al., 2021) and promotes GAS6 expression in phagocytes, thus promoting phagocytosis in an animal model of pneumonia (Nepal et al., 2019). In addition, STAT6 exerts neuroprotective effects by regulating the transformation of MMΦ from the M1 type to the M2 type and by promoting hematoma clearance in ICH (Xu et al., 2020b). We hypothesized that TSG6 promotes MMΦ polarization to the M2 type by activating the STAT6-GAS6 signaling pathway, thereby accelerating hematoma clearance and improving functional recovery in mice after ICH. This study thus provides potential intervention targets and new drug options for future ICH treatment.

## Methods

### Experimental design

This experimental study investigated TSG6’s role in ICH through four interconnected phases: first examining TSG6 expression patterns in both ICH patients and mouse models, then evaluating the therapeutic effects of recombinant human TSG6 through histological and behavioral assessments, followed by analyzing TSG6’s impact on macrophage/microglia polarization and phagocytic function, and finally elucidating the underlying STAT6/GAS6 signaling mechanism in hematoma absorption. The specific experimental flow chart is shown in **Additional Figure 1**.

### Intracerebral hemorrhage patient dataset

The gene expression profiles of patients with ICH were collected from the GEO database. The inclusion criteria were as follows: (1) mRNA expression profiles derived from humans; (2) at least two groups: ICH group and control group; and (3) no fewer than three samples per group. Correlation analysis was performed using an online data analysis and visualization tool (https://www.bioinformatics.com.cn).

### Animals

Specific pathogen–free male C57BL/6 mice (aged 8–10 weeks, weighing 22–23 g, *n* = 480) were provided by Charles River (Beijing, China, animal license No. SYXK (E) 2021-0057). Male mice were chosen to avoid hormonal variability and sex-specific immune differences that could affect experimental reliability and outcomes (Zajitschek et al., 2020; Tariq et al., 2023). The animal experiments were approved by the Laboratory Animal Center of Huazhong University of Science and Technology (Ethics approval No. TJH-201909015) on September 20, 2019. All animal experiments were carried out according to National Institutes of Health Guide for the Care and Use of Laboratory Animals guidelines (NIH Publications No. 8023, revised 1978). The mice were housed in a barrier system with access to an adequate food supply, a 12/12-hour light/dark schedule, a temperature of 20–26°C, and a humidity level of 50%–60%.

### Establishment of a mouse model of intracerebral hemorrhage

The ICH model was established as previously described (Liu et al., 2023b). Briefly, the mice were anesthetized using an intraperitoneal injection of 1% pentobarbital sodium (70 mg/kg; China National Pharmaceutical Group Chemical Products Co., Ltd, Shanghai, China). Then, the mice were fixed on a stereotactic device and injected with 0.6 μL of collagenase (Sigma Aldrich, St. Louis, MO, USA) into the caudate nucleus (anterior horn 0.5 mm, left side 2 mm, depth 3.5 mm) to induce ICH.

### Drug administration

rhTSG6 (R&D Systems, Minneapolis, MN, USA) was diluted in phosphate-buffered saline (PBS) to a concentration of 2 μg/μL and injected into the lateral ventricles (anterior horn 0.8 mm, left side 1.2 mm, depth 3 mm) of the mice 6 hours after ICH. AS1517499 (MedChemExpress, Princeton, NJ, USA), which can penetrate the blood–brain barrier (half maximal inhibitory concentration: 21 nM), was used to block STAT6 phosphorylation, as was fully validated in a previous study (Olde Heuvel et al., 2019). AS1517499 was dissolved in dimethyl sulfoxide (DMSO) to a concentration of 20 mg/mL. The AS1517499 solution was injected into the lateral ventricles of mice at a dose of 10 mg/kg) immediately following injection of collagenase into the caudate nucleus. The same volume of DMSO (0.05 mL/kg) was administered to mice in the control group.

### Cell culture and treatment

In vitro tests were conducted using BV2 microglial cells (Pricella Life Science & Technology Co., Ltd, Wuhan, China, Lot No. CL-0493, RRID:CVCL_0182), and hemin was used to simulate the ICH environment (Lv et al., 2023). A Cell Counting Kit-8 (CCK-8) assay (Dojindo, Tokyo, Japan) was performed to measure cell viability after adding different concentrations (0, 10, 20, 40, 60, and 80 µM) of hemin (Sigma Aldrich) to the growth medium and incubating for 24 hours. A hemin concentration (40 µM) that resulted in 50% cell viability was used for the following experiments. In addition, BV2 cells were transfected with different adenoviruses, including a TSG6 overexpression virus (OE-TSG6; GeneChem Co., Ltd., Shanghai, China), an overexpression control lentivirus (OE-Ctrl; GeneChem Co., Ltd), a TSG6 knockdown lentivirus (sh-TSG6, GeneChem Co., Ltd), and a knockdown control lentivirus (sh-Ctrl; GeneChem Co., Ltd), for 72 hours to overexpress or knock down TSG6 expression. The specific volume of lentivirus solution added was calculated on the basis of the number of cells, the multiplicity of infection, and the lentiviral titer, as follows: the amount of virus added per well (μL) = multiplicity of infection × number of cells/virus titer (Tu/mL) × 1000. After transfection, polybrene was added to a concentration of 5 μg/mL.

### Western blotting

Phenylmethanesulfonyl fluoride (100 mM), radioimmunoprecipitation assay lysis buffer containing phosphatase inhibitors, and a protease inhibitor cocktail (50×) were used to extract total proteins from brain tissues and cell samples. Following protein concentration measurement with a bicinchoninic acid assay kit (Beyotime, Shanghai, China), sodium dodecyl sulfate-polyacrylamide gel electrophoresis was used to separate the proteins, which were then transferred onto polyvinylidene fluoride membranes. After blocking in 5% bovine serum albumin for 1 hour at room temperature, the membranes were incubated overnight with the primary antibody at 4°C. The membrane was then incubated with the secondary antibody for 1.5 hours at room temperature. Detailed information regarding the antibodies that were used is provided in **Additional file 1**. A Bio-Rad Gel imaging system (Hercules, CA, USA) was used to visualize the protein bands, and protein expression levels were normalized to housekeeping proteins (GAPDH and tubulin) using ImageJ software (version 1.54i; National Institutes of Health, Bethesda, MD, USA; Schneider et al., 2012).

### Real-time polymerase chain reaction

Total RNA was extracted from homogenized brain tissue and cell samples using Trizol (Takara, Kyoto, Japan), and the concentration and purity of the total RNA were assessed. Next, a reverse transcription kit (Takara) was used to generate complementary DNA. A StepOne Real-Time PCR system (Applied Biosystems, Foster City, CA, USA) was used to perform real-time polymerase chain reaction (RT-PCR). The 2^–ΔΔCT^ technique was used to calculate relative gene expression levels (Arocho et al., 2006). The primer sequences used for RT-PCR were as follows: TSG6 forward (5′–3′), GGC TGG CAG ATA CAA GCT CA; TSG6 reverse (5′–3′), TCA AAT TCA CAT ACG GCC TTG G; GAPDH forward (5′–3′), CCT CGT CCC GTA GAC AAA ATG; and GAPDH reverse (5′–3′), TGA GGT CAA TGA AGG GGT CGT. The expression of TSG6 was normalized by GAPDH.

### Staining of brain tissue sections

The whole brain was perfused with PBS and 4% paraformaldehyde solution, then the entire brain was extracted and fixed in a 4% paraformaldehyde solution. After fixation, the brains were dehydrated, stained, embedded in paraffin, and sliced into 5-μm-thick sections surrounding the hematoma site. The sections were then mounted on microscope slides and dried for an hour at 60°C. Finally, the sections were subjected to immunohistochemistry, IF, TUNEL, Nissl staining, and iron staining, as described in previously published articles (Liu et al., 2023a, b; also available in **Additional file 1**).

### Measurement of hematoma volume

The entire brain was placed in a mold on ice and sliced into 1-mm-thick sections that were then placed flat in a 10-cm glass dish for measurement. The hematoma volume was calculated using ImageJ software as follows: hematoma volume (mm^3^) = Σ(frontal hematoma area + reverse hematoma area)/2 × 1 mm.

### Measurement of hemoglobin content

Brain tissue was placed in 400 μL of double-distilled water and homogenized, then centrifuged (4°C, 30 minutes, 2050 × *g*). Next, 20 µL of supernatant and 80 µL of prepared Drabkin’s reagent (Sigma Aldrich) were added to each well of a 96-well plate, and the mixture was incubated for 15 minutes at room temperature (20–25°C). To determine the hemoglobin concentration, the absolute optical density of the solution was measured at 540 nm.

### Neurobehavioral tests

Several neurobehavioral tests (Garcia score, corner turn test, adaptive removal test, cylinder test, and foot fault test) were used to assess neurological impairment in mice. With the exception of the pre-experiment, all behavioral tests were carried out 1 day prior to and 1, 3, 5, 7, and 14 days after ICH. Detailed information is provided in **Additional file 1**.

### Virus transfection

#### Adenovirus transfection of mice

Mouse brain tissue was transfected with adenoviruses (GeneChem Co., Ltd) to downregulate TSG6 expression (sh-TSG6). Transfection was performed 4 days before ICH was induced. Control AV (sh-ctrl) and sh-TSG6 AV (1.5E+7 PEU/μL, 3 μL) were injected at a rate of 0.3 μL/min into the caudate nucleus using the same technique as that described for collagenase injection earlier.

#### Lentivirus transfection of cells

The lentiviruses were constructed by Shanghai GeneChem Co., Ltd. The oe-TSG6 lentivirus induces TSG6 overexpression, while the sh-TSG6 lentivirus knocks down TSG6 expression. In addition, oe-ctrl and sh-ctrl (empty viral vector) virus infections were used as negative controls. After cells seeded into 6-well plates adhered and reached a density of roughly 40% to 50%, each well was filled with 1 mL of fresh, serum-free BV2 microglial cell culture media and fresh lentivirus and polybrene solutions and incubated for 4 hours in a cell incubator (37°C, 5% CO_2_). Next, 1 mL of complete culture medium containing 10% fetal bovine serum was then added to each well, and the plates were re-incubated for 24 hours, after which 2 mL of complete culture medium containing 10% fetal bovine serum was added. Following another 48 hours of incubation (for a total of 72 hours of lentivirus transfection), the cell culture medium was supplemented with 1.5 μg/mL puromycin, and the cells were cultured for an additional 24 hours.

### Cell phagocytosis

#### Red blood cell isolation and labeling

The retro-orbital venous plexus is a common site for blood collection in mice due to its easy accessibility, the ability to collect larger volumes, and the speed and consistency of blood collection (Van Herck et al., 2001; Gjendal et al., 2020). One of the mice described in the Animals subsection above was randomly selected for blood collection. Briefly, the mouse was anesthetized using an intraperitoneal injection of 1% pentobarbital sodium (70 mg/kg), and then 100 μL of blood was collected from the retro-orbital venous plexus with a capillary pipette and placed in a sterile anticoagulant tube (Wang et al., 2009). An equal volume of PBS was added, and the mixture was centrifuged (5 minutes, 411 × *g*), and the supernatant was removed. The pelleted RBCs were washed three times with Hanks’ balanced salt solution (without Ca^2+^ or Mg^2+^) and centrifuged (5 minutes, 411 × *g* the first two times and 10 minutes, 411 × *g* the third time). After the third centrifugation, the supernatant was removed, and the RBCs were resuspended in 50 mL of PBS containing 0.05 g bovine serum albumin.

pHrodo BioParticles conjugates (P35361, Thermofisher, Waltham, MA, USA) were diluted in PBS at a pH of 7.4 to a concentration of 1 mg/mL. For the *in vitro* phagocytosis experiments, 100 μL of the purified RBCs was co-cultured with pHrodo BioParticle conjugates at room temperature in the dark for 10 minutes.

#### Phagocytosis

Fluorescently labeled RBCs were incubated with BV2 microglial cells (RBC:microglia = 10:1) for 6 hours. Next, the cell growth medium was aspirated, and the cells were washed with PBS, fixed in paraformaldehyde, and subjected to IF staining (IBA1 + DAPI). The phagocytic index was calculated as the percentage of BV2 microglial cells containing RBCs in the total number of BV2 microglial cells.

### Statistical analysis

Normally distributed data are reported as mean ± standard deviation (SD), while non-normally distributed data are reported as medians and quantiles. Pearson correlation coefficient analysis was used to analyze the correlation between TSG6 and hematoma volume. Two-tailed Student’s *t*-test was used to compare two groups, and two-way or one-way analysis of variance with Tukey’s *post hoc* test was used to compare multiple groups. Statistical significance was defined as a *P*-value less than 0.05. Data analysis was performed and graphs were drawn using GraphPad Prism (version software 8.0 for Windows, GraphPad Software, Boston, MA, USA; www.graphpad.com).

## Results

### Tumor necrosis factor-α-stimulated gene 6 expression in patients with intracerebral hemorrhage

To investigate the relationship between TSG6 and ICH, gene expression profiles from patients with ICH and healthy donors were retrieved from the GEO public database. On the basis of the conditions specified in the Materials and Methods, a related dataset, GSE163256, was obtained from the GEO database. The dataset was publicly uploaded on January 9, 2021. We isolated monocytes and eosinophils from the peripheral blood and hematoma effluents of 399 patients with ICH and 14 healthy people (control group). Then, mRNA sequencing was performed to examine TSG6 expression profile in the different groups. The sample acquisition time was divided into 1–3 days (0–72 hours), 3–5 days (73–120 hours), and 5–7 days (121–171 hours) after ICH to detect dynamic changes in TSG6 expression in patients with ICH.

**Figure 1:**
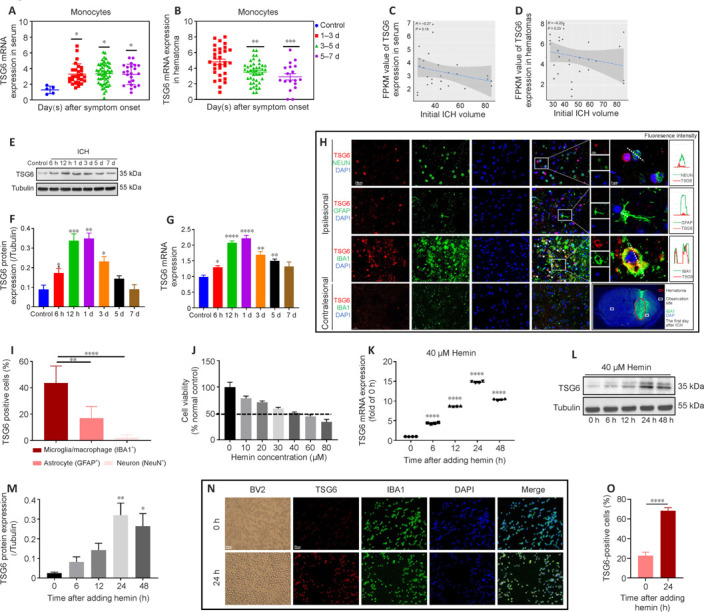
TSG6 expression in patients with ICH and mice subjected to ICH. (A, B) Detection of TSG6 mRNA in serum and hematoma effluent samples taken from patients with ICH at different time points. (C, D) Pearson correlation coefficient analysis between TSG6 mRNA levels in early hematoma effluent (FPKM value) and initial hematoma volume (mL). R indicates the degree of correlation, and P indicates the degree of significance. (E–G) TSG6 mRNA and protein expression levels in mouse brain tissue at different time points after ICH (*n* = 6). (H) IF staining for TSG6 (red, CoraLite594) (*n* = 3) on the 1^st^ day post-ICH showed that TSG6 mainly colocalized with the MMΦ marker IBA1 (green, CoraLite488) around the hematoma, with little colocalization with the astrocyte marker GFAP (green, CoraLite488) and no obvious colocalization with the neuron marker NeuN (green, CoraLite488). Scale bars: 5 µm, 10 μm, and 20 μm. (I) Quantitative analysis of TSG6 expression in microglia/macrophages (IBA1^+^), astrocytes (GFAP^+^), and neurons (NeuN^+^). (J) CCK-8 assay of BV2 microglial cells after 24 hours of treatment with different concentrations of hemin. (K–M) TSG6 mRNA and protein expression levels in cells at different time points (0, 6, 12, 24, and 48 hours) after hemin treatment (*n* = 3–4). (N) Morphological and immunofluorescence analyses of BV2 cells after 24 hours after hemin treatment (*n* = 3). The bright field images show that BV2 cells contracted after hemin treatment, and the immunofluorescence images show that TSG6 expression (red, CoraLite594) in BV2 cells (green, CoraLite488) increased after hemin treatment. (O) Quantitative analysis of TSG6 immunoreactivity in BV2 cells. Data are reported as mean ± SD. *P < 0.05, **P < 0.01, ***P < 0.001, ****P < 0.0001. One-way analysis of variance with Tukey’s *post hoc* test was used in A, B, F, G, I, K and M; two-tailed Student’s *t*-test was used in O. CCK-8: Cell Counting Kit-8; DAPI: 4′,6-diamidino-2-phenylindole; FPKM: fragments per kilobase of exon model per million mapped fragments; GFAP: glial fibrillary acidic protein; ICH: intracerebral hemorrhage; IBA1: ionized calcium binding adaptor molecule 1; MMΦ: microglia/macrophages; NeuN: neuronal nuclei; TSG6: tumor necrosis factor-α-stimulated gene 6.

TSG6 expression in monocytes was determined from the GSE163256 dataset. Serum monocytes from patients with ICH exhibited considerably greater levels of TSG6 mRNA than healthy controls. However, TSG6 mRNA expression in serum monocytes did not significantly change between different time periods after ICH (**Figure 1A**). By contrast, TSG6 mRNA expression in monocytes from hematoma effluent was significantly decreased at 3–5 days and 5–7 days compared with the level observed 1–3 days after ICH (**Figure 1B**). The relationship between TSG6 expression in monocytes from serum and hematoma effluent in the first 3 days post-ICH and the initial size of the hematoma was assessed by Pearson correlation coefficient analysis. The findings demonstrated a negative association between initial hematoma volume and TSG6 expression in both serum and hematoma effluent (**Figure 1C** and **D**).

### Tumor necrosis factor-α-stimulated gene 6 expression in *in vivo* and *in vitro* models of intracerebral hemorrhage

Next, the levels of TSG6 protein and mRNA in the lesional brain tissue of a collagenase-induced mouse model of ICH were analyzed at different time points. TSG6 protein expression was elevated 6 hours after ICH, peaked within 12 hours to 1 day, and then gradually declined by days 5 and 7 in comparison with the control group, as demonstrated by western blot (**Figure 1E** and **F**). Similarly, TSG6 mRNA expression increased beginning 6 hours after ICH and returned to normal by 7 days post-ICH (**Figure 1G**). IF staining conducted 1 day after ICH showed that TSG6 mainly colocalized with the MMΦ marker IBA1 around the hematoma, and the fluorescence density of TSG6 was consistent with that of IBA1; there was little colocalization with glial fibrillary acidic protein (an astrocyte marker), and no obvious colocalization with the neuronal nucleus marker NeuN (**Figure 1H** and **I**). Almost no TSG6 was expressed in the contralateral brain tissue. This indicates that TSG6 is mainly expressed in MMΦ around the hematoma in mice post-ICH. An *in vitro* ICH model was constructed by treating BV2 microglial cells with hemin. On the basis of its ability to induce a 50% decrease in cell viability in the CCK-8 assay, we selected 40 μM hemin for use in subsequent experiments (**Figure 1J**). TSG6 mRNA and protein expression in BV2 cells treated with 40 μM hemin was detected at different time points. Following 6 hours of hemin treatment, TSG6 mRNA and protein expression levels started to rise, peaked at about 24 hours, and then started to decline (**Figure 1K–M**). After 24 hours of hemin treatment, BV2 cell morphology changed, and IF confirmed the increase in TSG6 expression (**Figure 1N** and **O**).

### Tumor necrosis factor-α-stimulated gene 6 accelerates hematoma absorption in a mouse model of intracerebral hemorrhage

Hematoma formation was observed in the basal ganglion regions of mice 6 hours after collagenase injection, and the hematomas reached a maximum volume at 24 hours. Endogenous hematoma absorption gradually decreases the volume of the hematoma. Compared with the sham group, the hematoma size decreased substantially between 3 and 5 days after ICH; however, the hematoma volume did not change significantly at 7 days (**Additional Figure 2A** and **B**).

**Figure 2:**
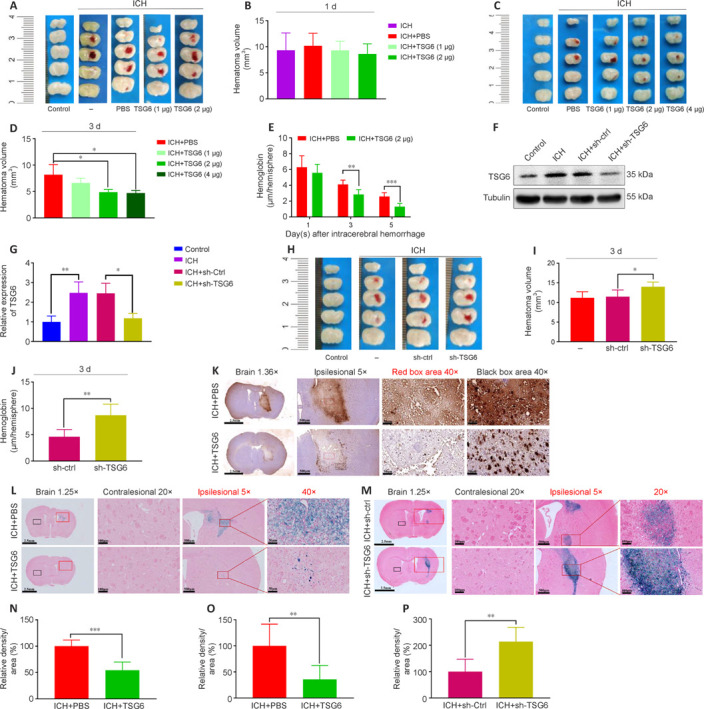
Effect of TSG6 on hematoma volume and iron deposition in a mouse model of ICH. (A, B) Representative images and statistical analysis of hematoma volume 1 day post-ICH. No substantial change was observed in hematoma volumes among mice subjected to ICH and treated with various doses (1 and 2 μg) of rhTSG6 and those in the PBS group on the first day following ICH. (C, D) Representative images and statistical analysis of hematoma volume 3 days post-ICH. (E) Hemoglobin content of the affected brain tissue on the 1^st^, 3^rd^, and 5^th^ days after ICH. (F, G) After injection with sh-TSG6 adenovirus, TSG6 expression in the brain tissue of mice subjected to ICH was detected by western blot. (H, I) Representative images (front and back of brain sections) and statistical analysis of hematoma volume on the 3^rd^ day after ICH in mice. Hematoma volume increased after reducing TSG6 expression in mouse brain tissue via transfection with the sh-TSG6 adenovirus. (J) Hemoglobin content in the brain tissue of mice from the ICH + sh-ctrl and ICH + sh-TSG6 groups on the 3^rd^ day after ICH. (K–P) Compared with the ICH + PBS group, there was significantly less iron deposition in the ICH + TSG6 group on the 3^rd^ (K, N) and 14^th^ day following ICH (L, O). In contrast to the ICH + sh-ctrl group, there was much greater iron deposition in the ICH + sh-TSG6 group on the 14^th^ day following ICH (M, P). Scale bars: 50 μm, 500 μm, and 2.5 mm. Data are presented as mean ± SD (*n* = 3–6). *P < 0.05, **P < 0.01, ***P < 0.001. One-way analysis of variance with Tukey’s *post hoc* test was used in B, D, E, G and I; two-tailed Student’s *t*-test was used in J and N–P. ICH: Intracerebral hemorrhage; PBS: phosphate-buffered saline; TSG6: tumor necrosis factor-α-stimulated gene 6.

Six hours after collagenase injection, rhTSG6 (1 or 2 μg) was injected into the lateral ventricles of ICH mice to assess the therapeutic efficacy of TSG6. No substantial change in hematoma volume was observed between mice treated with various doses of rhTSG6 and mice treated with PBS on the first day following ICH (**Figure 2A** and **B**). Subsequently, the observation time was extended to the 3rd day after ICH, and a higher-dose rhTSG6 treatment group (4 μg) was added. The hematoma volume in the ICH + TSG6 (2 μg) and ICH + TSG6 (4 μg) groups was considerably smaller than that in the ICH + PBS group. Furthermore, no discernible difference in hematoma volume was noted between the ICH + TSG6 (2 μg) and ICH + TSG6 (4 μg) groups (**Figure 2C** and **D**). Therefore, we selected 2 μg rhTSG6 as the therapeutic dose for use in subsequent experiments. Detection of hemoglobin content in mouse brain tissue showed that, on days 3 and 5 following ICH, the ICH + rhTSG6 group had a considerably lower hemoglobin content than the ICH + PBS group (**Figure 2E**).

Mouse brains were transfected with adenoviruses to inhibit TSG6 expression to further validate the impact of TSG6 on hematoma absorption in mice subjected to ICH. TSG6 protein expression in the ICH + sh-TSG6 group was substantially reduced to approximately one-third of that seen in the ICH + sh-ctrl group (**Figure 2F** and **G**). The hematoma volume in the ICH + sh-TSG6 group was larger than that in the ICH + sh-ctrl group 3 days after ICH (**Figure 2H** and **I**). Similarly, the ICH + sh-TSG6 group exhibited a significant increase in hemoglobin content compared with the ICH + sh-ctrl group (**Figure 2J**). These results indicate that TSG6 expression was negatively correlated with hematoma volume and hemoglobin content, which is consistent with our findings from patients with ICH, as shown in **Figure 1C** and **D**. Therefore, TSG6 is a potential therapeutic target for accelerating hematoma absorption after ICH.

Three days post-ICH, HE staining showed significant MMΦ infiltration surrounding the hematoma (**Additional Figure 3A**). The ICH + rhTSG6 group exhibited a substantial increase in MMΦ phagocytosis of RBCs in the brain tissue compared with the ICH + PBS group (**Additional Figure 3B**). In contrast to the ICH + sh-ctrl group, the ICH + sh-TSG6 group exhibited a much lower number of MMΦ engulfing erythrocytes (**Additional Figure 3C** and **D**). This result implies that TSG6 promotes MMΦ phagocytic activity in the brain tissue of mice subjected to ICH.

As described above, hematoma size decreased significantly 3 and 5 days after ICH due to endogenous hematoma absorption, but no significant change in hematoma volume was observed at 7 days post-ICH. Because hematomas clearance is challenging to observe with the naked eye over long time periods, we used an iron staining kit to evaluate long-term hematoma clearance because a recent study showed that iron, a hematoma metabolite, persists post-ICH (Magid-Bernstein et al., 2022). Compared with the ICH + PBS group, there was significantly less iron deposition in the ICH + TSG6 group on days 3 (**Figure 2K** and **N**) and 14 following ICH (**Figure 2L** and **O**). In contrast to the ICH + sh-ctrl group, iron deposition in the ICH + sh-TSG6 group was much higher on the day 14 following ICH (**Figure 2M** and **P**). Thus, rhTSG6 dramatically reduced iron deposition in mice subjected to ICH, as evidenced by the negative correlation observed between iron deposition and TSG6 expression.

### Tumor necrosis factor-α-stimulated gene 6 ameliorates neuronal injury and promotes neurobehavioral recovery in a mouse model of intracerebral hemorrhage

**Figure 3:**
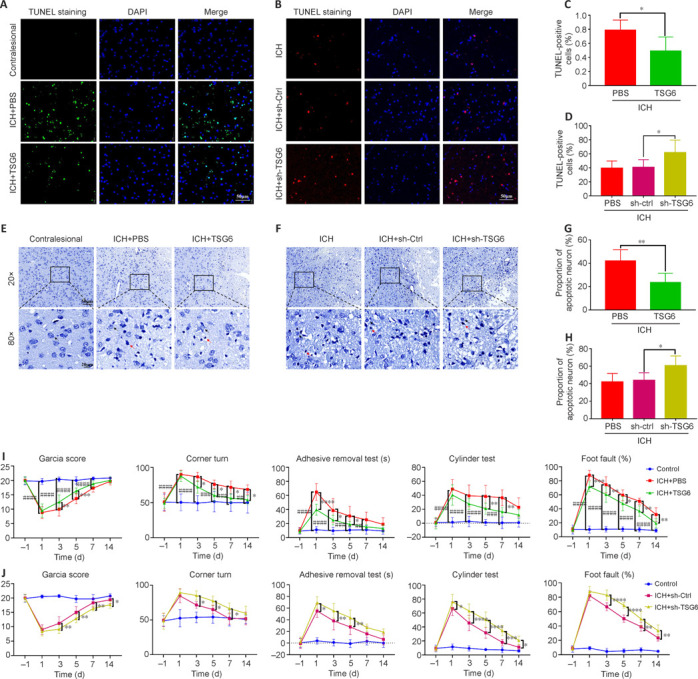
TSG6 protects brain cells. (A–D) TUNEL staining demonstrated that, in the ICH + TSG6 group, the proportion of apoptotic cells surrounding the hematoma was significantly lower than in the ICH + PBS group (A, C). By contrast, there were significantly more apoptotic cells in the ICH + sh-TSG6 group than in the ICH + sh-ctrl group (B, D). (E–H) Nissl staining showed that the ICH + PBS group exhibited fewer Nissl bodies surrounding the hematoma, along with shrunken and deformed cell bodies. In the ICH + TSG6 group, however, more clear and intact Nissl bodies were visible around the hematoma (E). Statistical analysis showed that the ICH + TSG6 group exhibited a much lower number of abnormal neurons than the ICH + PBS group (G). Correspondingly, a significant increase in the number of abnormal neurons was found in the ICH + sh-TSG6 group in comparison with the ICH + sh-ctrl group (F, H). (I, J) One day before ICH and 1, 3, 5, 7, and 14 days after ICH, the three groups of mice were subjected to neurobehavioral testing (*n* = 6). (I) The control and ICH + PBS groups were compared, and the ICH + PBS and ICH + TSG6 groups were compared. (J) The ICH + sh-ctrl and ICH + sh-TSG6 groups were compared. All data are reported as mean ± SD. ##*P* < 0.01, ###*P* < 0.001, ####*P* < 0.0001, *vs.* control group; *P < 0.05, **P < 0.01, ***P < 0.001, ****P < 0.0001, *vs*. ICH + PBS group or ICH + sh-ctrl group. Two-tailed Student’s *t*-test was used in C and G; one-way analysis of variance with Tukey’s *post hoc* test was used in D and H; two-way analysis of variance with Tukey’s *post hoc* test was used in I and J. Ctrl: Control; DAPI: 4′,6-diamidino-2-phenylindole; ICH: intracerebral hemorrhage; PBS: phosphate-buffered saline; TSG6: tumor necrosis factor-α-stimulated gene 6; TUNEL: transferase-mediated dUTP nick-end labeling.

Using TUNEL staining, we assessed brain cell apoptosis in each group. The proportion of apoptotic cells surrounding the hematoma was significantly lower in the ICH + TSG6 group than in the ICH + PBS group (**Figure 3A** and **C**); by contrast, the number of apoptotic cells in the ICH + sh-TSG6 group was significantly higher than that in the ICH + sh-ctrl group (**Figure 3B** and **D**). This indicates that, in mice subjected to ICH, TSG6 dramatically lowers the number of apoptotic cells surrounding hematomas. Furthermore, Nissl staining showed fewer Nissl bodies surrounding the hematoma, along with shrunken and deformed cell bodies in the ICH + PBS group than in the ICH + TSG6 group. In the ICH + TSG6 group, however, more clear and intact Nissl bodies were visible around the hematoma (**Figure 3E**). Statistical analysis showed that the ICH + TSG6 group had a much lower number of abnormal neurons than the ICH + PBS group (**Figure 3G**). Correspondingly, a significant increase in the number of abnormal neurons was observed in the ICH + sh-TSG6 group in comparison with the ICH + sh-ctrl group (**Figure 3F** and **H**). This finding indicates that inhibiting TSG6 expression increases the number of abnormal neurons, while rhTSG6 treatment improves survival of normal neurons.

To verify the protective effect of TSG6 in mice subjected to ICH, a set of functional behavior tests was carried out. The adapted removal test showed that, compared with mice in the ICH+PBS group, mice in the ICH + TSG6 group took less time to remove the adhesive on the 1^st^, 3^rd^, and 5^th^ days after ICH; the corner turn test showed that the right turn ratio of the mice in the ICH + TSG6 group was decreased on the 3^rd^, 5^th^, 7^th^, and 14^th^ day after ICH; the modified Garcia score showed a significant increase in the ICH + TSG6 group score after ICH; and the cylinder test and foot fault test showed that the mice in ICH + TSG6 group exhibited a reduced rate of using the right front paw and a reduced number of left limb drops after ICH (**Figure 3I**). Consistent with these findings, recovery of neurological function occurred more slowly in mice in the ICH + sh-TSG6 group in comparison with mice in the ICH + sh-ctrl group (**Figure 3J**). These results indicate that early TSG6 levels predict symptom severity and recovery rate in mice subjected to ICH, and that rhTSG6 treatment can significantly promote behavioral recovery in these mice.

### Tumor necrosis factor-α-stimulated gene 6 induces microglia/macrophages polarization in a mouse model of intracerebral hemorrhage

**Figure 4:**
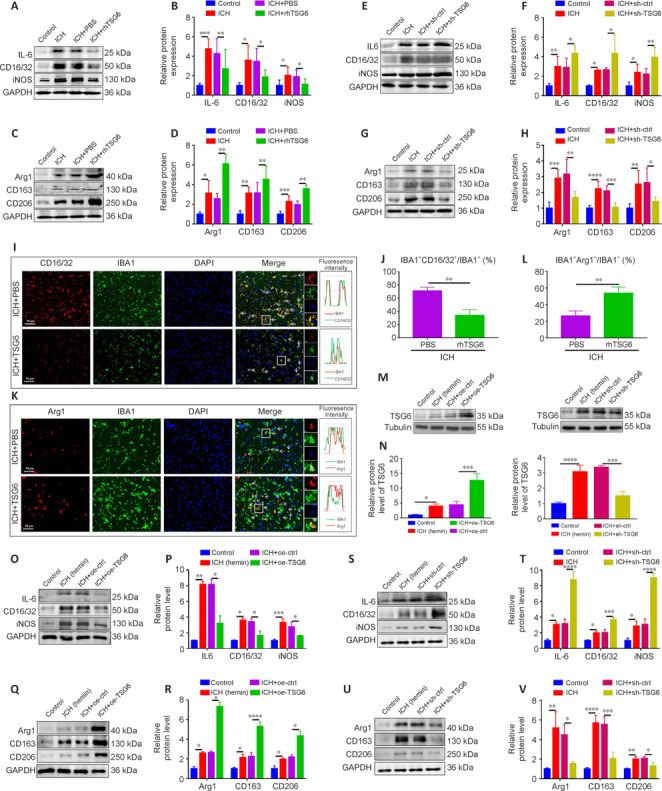
Effects of endogenous TSG6 and exogenous rhTSG6 on the expression of inflammatory factors *in vivo* and *in vitro* ICH models, respectively. (A–D) Western blot analysis of the effect of exogenous rhTSG6 treatment on the expression of M1-type inflammatory factors (IL6, CD16/32, and iNOS) and M2-type inflammatory factors (Arg1, CD163, and CD206) in mouse brain tissue. (E–H) Western blot detection of the effect of sh-TSG6 adenovirus transfection on the expression of M1 and M2 inflammatory factors in mouse brain tissue. (I–L) IF detection of the effect of TSG6 on CD16/32 and Arg1 expression in the brain tissue of mice subjected to ICH. The ICH+TSG6 group exhibited a lower proportion of M1-type IBA1^+^CD16/32^+^ cells (I, J) and a higher proportion of M2-type Iba1^+^Arg1^+^ cells (K, L) in comparison with the ICH + PBS group. Red (CoraLite594) indicates Arg1^+^ or CD16/32^+^ cells; green (CoraLite488) indicates IBA1^+^ cells (IBA1 is a MMΦ marker). The white arrow indicates MMΦ that simultaneously expressed IBA1 and Arg1 or CD16/32 protein. The white box indicates typical cells whose fluorescence intensity was analyzed. The graph on the right side of the immunofluorescence image shows fluorescence intensity analysis of the selected typical cells using ImageJ software. Scale bars: 5 μm and 50 μm. (M, N) Western blot detection of oe-TSG6 lentivirus transfection efficiency *in vitro*. (O–V) The effect TSG6 overexpression (oe-TSG6) and TSG6 knockdown (sh-TSG6) on the expression levels of M1 and M2 inflammatory factors in BV2 microglial cells. All data are reported as mean ± SD (*n* = 3–6). *P < 0.05, **P < 0.01, ***P < 0.001, ****P < 0.0001. One-way analysis of variance with Tukey’s *post hoc* test was used in B, D, F, H, N, P, R, T and V; two-tailed Student’s *t*-test was used in J and L. Arg1: Arginase1; CD16/32: cluster of differentiation16/32; CD163: cluster of differentiation163; CD206: cluster of differentiation206; DAPI: 4′,6-diamidino-2-phenylindole; GAPDH: glyceraldehyde 3-phosphate dehydrogenase; IBA1: ionized calcium binding adaptor molecule 1; ICH: intracerebral hemorrhage; IL-6: interleukin-6; iNOS: inducible nitric oxide synthase; MMΦ: microglia/macrophages; PBS: phosphate-buffered saline; rhTSG6: recombinant human TSG6; TSG6: tumor necrosis factor-α-stimulated gene 6.

To determine whether TSG6 influences MMΦ polarization in mice subjected to ICH, the protein levels of M1-type (IL6, CD16/32, and iNOS) and M2-type (Arg1, CD163, and CD206) inflammatory factors in brain tissue from the different groups were measured on the 3rd day after ICH. Compared with the ICH + PBS group, the ICH + rhTSG6 group showed a decrease in M1-type inflammatory factor levels and an increase in M2-type inflammatory factor levels (**Figure 4A–D**); by contrast, the ICH + sh-TSG6 group showed an increase in M1-type inflammatory factor levels and a decrease in M2-type inflammatory factor levels in comparison with the ICH + sh-ctrl group (**Figure 4E–H**). Furthermore, IF staining demonstrated that the ICH + rhTSG6 group exhibited a higher proportion of M2-type IBA1^+^ ARG1^+^ cells and a lower proportion of M1-type IBA1^+^ CD16/32^+^ cells in comparison with the ICH + PBS group (**Figure 4I–L**). This finding suggests that, in mice with ICH, endogenous TSG6 expression may also influence the expression of M1/M2-type inflammatory factors, and that rhTSG6 treatment may encourage MMΦ polarization from the M1 type toward the M2 type in brain tissue.

Microglia, the primary phagocytes in the brain, are crucial for removing hematomas post-ICH (Xue and Yong, 2020). As described above, we stimulated BV2 cells with 40 μM hemin to create an *in vitro* model of ICH. Next, the BV2 microglial cells were transfected with several lentiviruses to either up- or downregulate TSG6 expression. BV2 cells treated with hemin and transfected with lentivirus overexpressing TSG6 are denoted as ICH + oe-TSG6, and BV2 cells treated with hemin and transfected with lentivirus downregulating TSG6 are denoted as ICH + sh-TSG6. First, western blot detection of TSG6 was used to evaluate the transfection efficiency. TSG6 expression was not affected by transfection with either the oe-ctrl lentivirus or the sh-ctrl control virus. TSG6 expression in the ICH + oe-TSG6 group was significantly (almost five times) higher than that in the ICH + oe-ctrl group. TSG6 expression in the ICH + sh-TSG6 group was substantially (about two-thirds) lower than that in the ICH + sh-ctrl group (**Figure 4M** and **N**). This result suggests that lentivirus transfection *in vitro* effectively regulated TSG6 expression in BV2 microglial cells.

Next, the levels of proteins secreted by M1 microglia (IL6, CD16/32, and iNOS) and M2 microglia (ARG1, CD163, and CD206) were detected. Compared with the control group, M1 and M2 inflammatory factor expression levels were significantly higher in the ICH + oe-ctrl and ICH + sh-ctrl groups (**Figure 4O–V**). The ICH + oe-TSG6 group exhibited an increase in M2-type inflammatory factor expression levels and a decrease in M1-type inflammatory factor expression levels compared with the ICH + oe-ctrl group (**Figure 4O–R**); by contrast, the ICH + sh-TSG6 group exhibited a significant decrease in M2-type inflammatory factors and an increase in M1-type inflammatory factors (**Figure 4S–V**). This finding suggests that TSG6, which is expressed by activated microglia *in vitro*, can promote MMΦ transformation from the M1 to the M2 phenotype.

### Tumor necrosis factor-α-stimulated gene 6 improves microglia/macrophages phagocytic function in a mouse model of intracerebral hemorrhage

**Figure 5:**
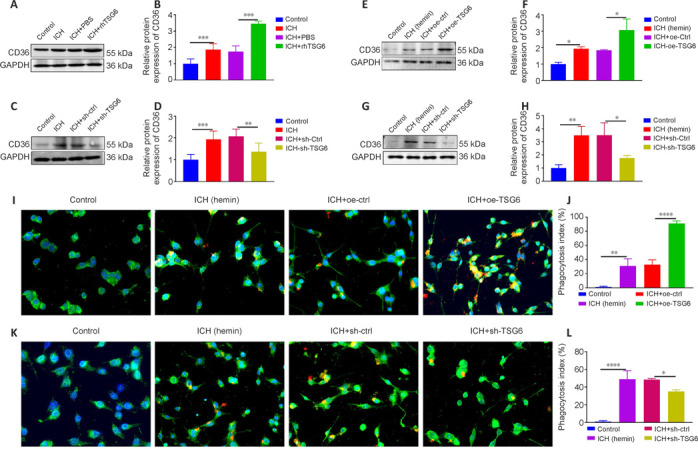
Effect of TSG6 on expression of the phagocytosis-associated protein CD36. (A–D) The effect of exogenous rhTSG6 or TSG6 inhibition (sh-TSG6) on CD36 expression in mouse brain tissue (*n* = 6). (E–H) The effect of TSG6 overexpression or inhibition on CD36 expression in BV2 microglial cells (*n* = 3). (I–L) *In vitro* phagocytosis experiments showing that no stained RBCs were found in control cells, while the number of RBCs in the ICH + oe-TSG6 group was significantly increased in comparison with the ICH + oe-ctrl group (I, J). Compared with ICH + sh-ctrl group, the proportion of microglia phagocytosing erythrocytes in the ICH + sh-TSG6 group was significantly decreased (K, L) (*n* = 3). RBCs (red), BV2 microglia (green, CoraLite488), DAPI (blue). Yellow indicates BV2 microglia engulfing red blood cells. Scale bars: 20 and 200 μm. All data are reported as mean ± SD (*n* = 3–6). *P < 0.05, **P < 0.01, ***P < 0.001, ****P < 0.0001. One-way analysis of variance with Tukey’s *post hoc* test was used for all statistical comparisons. CD36: Cluster of differentiation36; GAPDH: glyceraldehyde 3-phosphate dehydrogenase; ICH: intracerebral hemorrhage; PBS: phosphate-buffered saline; rhTSG6: recombinant human TSG6; TSG6: tumor necrosis factor-α-stimulated gene 6.

To investigate the impact of TSG6 on MMΦ phagocytic function, expression of the phagocytic-related protein CD36 was measured 3 days post-ICH in each group. Compared with the Control group, CD36 expression in the ICH group was significantly increased; similarly, compared with the ICH + PBS group, CD36 expression in the ICH + TSG6 group was significantly increased (**Figure 5A** and **B**). CD36 expression in the ICH + sh-TSG6 group was substantially lower than that in the ICH + sh-ctrl group (**Figure 5C** and **D**). To clarify the impact of TSG6 expression on phagocytosis by activated microglia, CD36 expression was evaluated in an *in vitro* model of ICH. CD36 expression levels in the ICH group were substantially higher than those in the control group; additionally, CD36 levels in the ICH + oe-TSG6 group were significantly higher than those in the ICH+oe-ctrl group (**Figure 5E** and **F**). CD36 expression levels were considerably lower in the ICH + sh-TSG6 group compared with the ICH + sh-ctrl group (**Figure 5G** and **H**). The findings show that CD36 expression correlates with TSG6 expression, suggesting that TSG6 expression by activated microglia influences the expression of CD36, a protein linked to phagocytosis (Grajchen et al., 2020), consequently influencing phagocytosis by microglia.

To visually observe the effect of TSG6 on MMΦ phagocytic function, we conducted *in vitro* phagocytic experiments. No stained RBCs were found in untreated BV2 microglial cells; however, after hemin treatment, the BV2 cells exhibited larger bulges, and the proportion of cells that had phagocytosed RBCs was higher than that in the control group. The proportion of BV2 microglial cell phagocytosing erythrocytes in the ICH + oe-TSG6 group was significantly increased comparison with the ICH + oe-ctrl group (**Figure 5I** and **J**). Compared with the ICH + sh-ctrl group, the proportion of microglia phagocytosing erythrocytes in the ICH + sh-TSG6 group was significantly decreased (**Figure 5K** and **L**). These findings show that endogenous TSG6 expression levels promote microglia-mediated phagocytosis of RBCs.

### STAT6 is a key downstream signaling molecule mediating the beneficial effects of TSG6 in a mouse model of intracerebral hemorrhage

**Figure 6:**
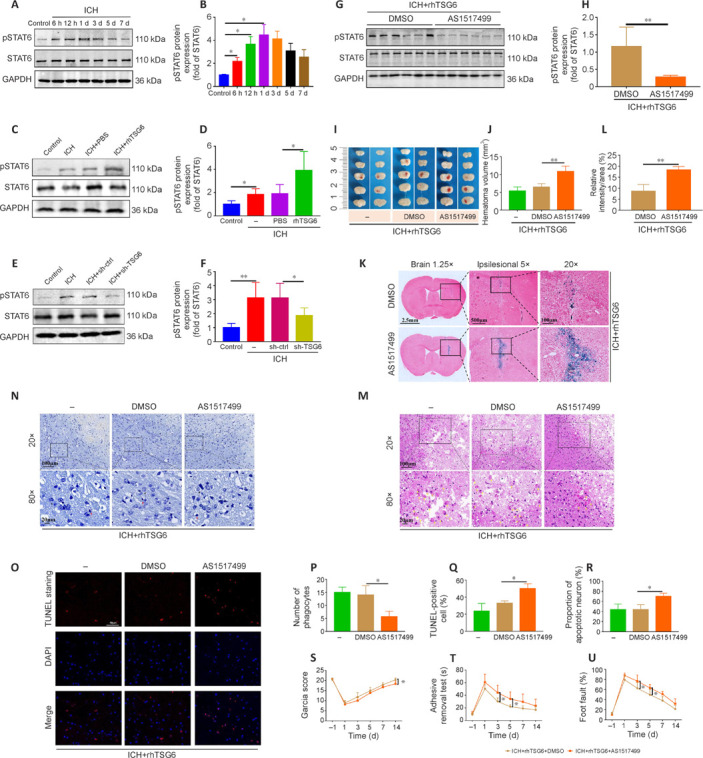
Role of STAT6 in TSG6 treatment of mice subjected to ICH. (A, B) Western blot detection of pSTAT6/STAT6 expression at different time points after ICH. (C–F) Western blot detection of pSTAT6/STAT6 expression in mouse brain tissue after rhTSG6 treatment or transfection with the sh-TSG6 adenovirus. (G, H) Western blot detection of pSTAT6/STAT6 in the brain tissue of mice in the DMSO and AS1517499 groups treated with rhTSG6 on the 3^rd^ day after ICH. (I–L) The small molecule AS1517499 reversed the beneficial effect of TSG6 on hematoma clearance in mice with ICH. (I, J) Representative images and statistical analysis of hematoma volume on the 3^rd^ day after ICH. (K, L) The levels of iron deposition in the AS1517499 group were considerably higher than those in the DMSO group on the 14^th^ day after ICH. (M, P) The number of MMΦ around the hematoma in the blank, DMSO, and AS1517499 groups, as detected by hematoxylin-eosin staining. TSG6-mediated promotion of RBC phagocytosis by MMΦ was impaired by inhibiting STAT6 phosphorylation with AS1517499. The yellow arrows indicate MMΦ engulfing RBCs. (N, R) Nissl staining of abnormal neurons around the hematoma in brain sections from the blank group, DMSO group, and AS1517499 group. More degenerated neurons were observed in the AS1517499 group than in the DMSO groups. The red arrows indicate abnormal neurons, while the black arrows indicate normal neurons. (O, Q) TUNEL of apoptotic cells around the hematoma in the blank, DMSO, and AS1517499 groups. More apoptotic cells were observed in the AS1517499 group than in the DMSO groups. Red indicates TUNEL-positive cells and blue represents DAPI. (S–U) One day before ICH and 1, 3, 5, 7, and 14 days after ICH, mice were subjected to neurobehavioral tests. The ICH + rhTSG6 + DMSO and ICH + rhTSG6 + AS1517499 groups were compared. Scale bars: 20 μm, 25 μm, and 50 μm. All data are reported as mean ± SD (*n* = 3–6). *P < 0.05, **P < 0.01. Two-way analysis of variance with Tukey’s *post hoc* test was used in S–U; one-way analysis of variance with Tukey’s *post hoc* test was used in B, D, J, F, P–R; two-tailed Student’s *t*-test was used in H and L. DAPI: 4′,6-Diamidino-2-phenylindole; DMSO: dimethyl sulfoxide; GAPDH: glyceraldehyde 3-phosphate dehydrogenase; ICH: intracerebral hemorrhage; PBS: phosphate-buffered saline; pSTAT6: phosphorylated STAT6; MMΦ: microglia/macrophages; RBC: red blood cell; rhTSG6: recombinant human TSG6; STAT6: signal transducer and activator of transcription 6; TUNEL: transferase-mediated dUTP nick-end labeling.

Dynamic changes in STAT6 phosphorylation following ICH were detected by western blot. Phosphorylated STAT6 (pSTAT6) levels were considerably higher 6 hours following ICH than in the control group, and peaked at 1 day (**Figure 6A** and **B**). Following rhTSG6 treatment, pSTAT6 levels increased dramatically (**Figure 6C** and **D**), whereas pSTAT6 levels were significantly lower in the ICH + sh-TSG6 group in comparison with the ICH + sh-Ctrl group (**Figure 6E** and **F**). To explore whether STAT6 phosphorylation levels influence the impact of TSG6 on hematoma absorption in mice subjected to ICH, the selective STAT6 phosphorylation inhibitor AS1517499 (10 mg/kg) was administered via the lateral ventricles to block STAT6 phosphorylation. First, STAT6 phosphorylation levels were detected in mice subjected to ICH and treated with rhTSG6 to verify inhibitory efficiency of STAT6 phosphorylation efficiency by AS1517499 or DMSO. STAT6 phosphorylation levels in the AS1517499 treatment group were reduced by about one-fourth compared with the DMSO group (**Figure 6G** and **H**). Next, the mice were grouped into the following three groups: ICH + rhTSG6 (blank group), ICH + rhTSG6 + DMSO (DMSO group), and ICH + rhTSG6 + AS1517499 (AS1517499 group).

The hematoma volume in the DMSO group was much smaller than that in the AS1517499 group (**Figure 6I** and **J**). The amount of iron deposition in the AS1517499 group was considerably higher than that in the DMSO group 14 days after ICH (**Figure 6K** and **L**). On the third day post-ICH, HE staining of brain tissue sections demonstrated that the TSG6-induced promotion of RBC phagocytosis by MMΦ was decreased by treatment with AS1517499 (**Figure 6M** and **P**). In addition, TUNEL showed that the AS1517499 group exhibited more apoptotic cells compared with the DMSO group (**Figure 6O** and **Q**). Furthermore, Nissl staining detected more degenerated neurons in the AS1517499 group compared with the DMSO group (**Figure 6N** and **R**). Behavioral tests showed that, in comparison with the DMSO group, the AS1517499 group exhibited a decrease in Garcia score on the 14^th^ day post-ICH, a longer adhesive removal time, and an increase in foot fault ratio on the 3^rd^ day post-ICH (**Figure 6S–U**). These results show that inhibiting STAT6 phosphorylation via treatment with AS1517499 decreases the ability of TSG6 to promote hematoma absorption in mice subjected to ICH, indicating that STAT6 is a key downstream signaling molecule of TSG6.

### The effect of TSG6/STAT6 on downstream targets GAS6 and AXL/MERTK in a mouse model of intracerebral hemorrhage

**Figure 7:**
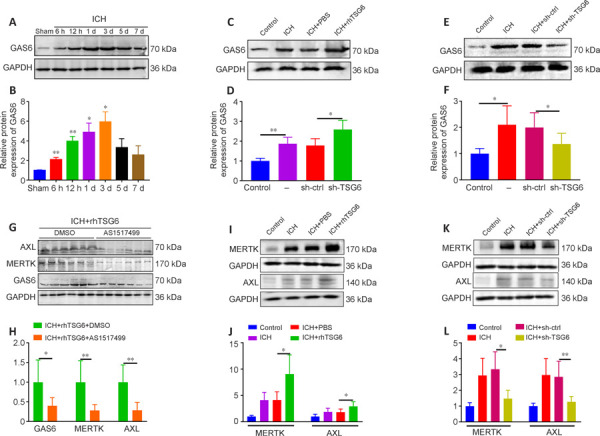
Effect of TSG6/STAT6 on downstream targets GAS6 and AXL/MERTK in a mouse model of ICH. (A, B) Western blot detection of GAS6 protein expression in brain tissue at different time points after ICH. (C–F) Western blot detection of GAS6 protein expression in mouse brain tissue after rhTSG6 treatment or transfection with sh-TSG6 AV. (G, H) Western blot detection of GAS6, MERTK, and AXL protein expression in the DMSO and AS1517499 groups. (I–L) Western blot detection of GAS6, MERTK, and AXL protein expression in mouse brain tissue after rhTSG6 treatment or transfection with sh-TSG6 AV. Data are reported as mean ± SD (*n* = 6). *P < 0.05, **P < 0.01. One-way analysis of variance with Tukey’s *post hoc* test was used in B, D, F, J and L; two-tailed Student’s *t*-test was used in H. AV: Adenovirus; AXL: Anexelekto; DMSO: dimethyl sulfoxide; GAPDH: glyceraldehyde 3-phosphate dehydrogenase; GAS6: growth arrest specific protein 6; ICH: intracerebral hemorrhage; MERTK: Mer receptor tyrosine kinase; PBS: phosphate-buffered saline; rhTSG6: recombinant human TSG6; TSG6: tumor necrosis factor-α-stimulated gene 6.

Considering that GAS6 is an extremely important downstream target of STAT6 for phagocytosis in other disease models (Nepal et al., 2019; Soliman et al., 2023), we hypothesized that GAS6 is also the key target of STAT6 in promoting hematoma clearance. To test this, we first measured GAS6 expression levels at various intervals following ICH. GAS6 expression was elevated 6 hours after ICH and peaked on the 3^rd^ day post-ICH (**Figure 7A** and **B**). Treatment with rhTSG6 significantly increased GAS6 expression (**Figure 7C** and **D**), while GAS6 expression in the ICH + sh-TSG6 group was significantly lower than that in the ICH + sh-ctrl group (**Figure 7E** and **F**). In addition, in mice subjected to ICH and treated with rhTSG6, there was a significant decrease in GAS6 expression in the AS1517499 group in comparison with the DMSO group (**Figure 7G** and **H**). These results indicate that GAS6 is the key downstream target of TSG6/STAT6 involved in promoting phagocytosis in a mouse model of ICH. Because GAS6 is an intermediate protein connecting PtdSer and the phagocytosis-related protein MERTK/AXL (Jiménez-García et al., 2022), we speculated that it may promote hematoma clearance after ICH by upregulating MERTK and AXL protein levels. Indeed, MERTK and AXL expression levels in the ICH + rhTSG6 group were substantially higher than those in the ICH + PBS group (**Figure 7I** and **J**). Both MERTK and AXL expression levels were considerably lower in the ICH + sh-TSG6 group than in the ICH + sh-ctrl group (**Figure 7K** and **L**). These results indicate that TSG6, as an upstream signal, can increase MERTK and AXL expression levels in with a mouse model of ICH. In addition, inhibiting STAT6 phosphorylation via treatment with AS1517499 decreased MERTK and AXL expression levels (**Figure 7G** and **H**). These results indicate that GAS6 regulates the expression of the phagocytosis-related receptors MERTK and AXL, thereby promoting rapid hematoma absorption after ICH in a mouse model.

## Discussion

This study comprehensively illustrated the function of TSG6 in hematoma absorption post-ICH and identified the potential molecular pathways by which TSG6 may facilitate MMΦ phagocytosis. By enhancing activation of the STAT6/GAS6 signaling pathway in the brain tissue of mice subjected to ICH, TSG6 promotes MMΦ phagocytosis of RBC, thereby accelerating hematoma absorption and improving post-ICH prognosis. Specifically, TSG6 promotes MMΦ polarization to the M2 phenotype and enhances the phagocytic function of microglia. To our knowledge, our study is the first to demonstrate the critical function of the STAT6/GAS6 signaling pathway in promoting hematoma clearance after ICH. In conclusion, the in-depth understanding of the pathophysiological events that occur after ICH provided by our study suggest potential treatment targets for ICH.

Although different types of drugs, such as antihypertensive agents, dehydrating agents, free radical scavengers, and neuroprotective agents, have been used for ICH treatment, their usage limitations and unknown interactions make their clinical efficacy unsatisfactory (An et al., 2017). Therefore, there is an urgent need to identify more effective therapeutic options. Although several studies have reported that TSG6 secreted by MSCs has a protective effect in ICH models (Chen et al., 2015; Tang et al., 2021), very little is known about the direct effects of endogenous TSG6, particularly its role in hematoma absorption. Our study showed that TSG6 was expressed in both patients with ICH and mice subjected to ICH, and that TSG6 expression increased and peaked shortly after collagenase injection in a mouse model of ICH. In addition, on the basis of GEO dataset analysis, we found a negative correlation between TSG6 expression in the early stage of ICH and initial hematoma volume, indicating that endogenous TSG6 may decrease hematoma volume after ICH. The lack of significant differences in our study may be because of the small sample size, differences in the time points at which samples were collected, and the high heterogeneity of online datasets. In the future, clinical studies should set strict specimen collection times and use appropriate sample sizes to explore the association between TSG6 levels and hematoma clearance rate.

The neuroprotective effects of TSG6 may primarily be mediated by its regulation of MMΦ polarization (Xu et al., 2020a). In our study, direct injection of TSG6 into the brain tissue effectively inhibited apoptosis, improved neuronal function, and promoted early functional recovery in mice. In addition, TSG6 function was confirmed by injecting adenoviruses to inhibit its expression. TSG6 has been proposed to be a novel predictor of cardiac function and myocardial fibrosis prognosis in patients with dilated cardiomyopathy-induced heart failure; it is also a 3-month prognostic marker for patients with non-cardiogenic acute ischemic stroke (Qu et al., 2022). The adverse effects of inhibiting TSG6 expression in our mouse model of ICH suggest that TSG6 could be used to predict the behavioral prognosis in patients with ICH. However, this needs to be confirmed through clinical research.

TSG6 produced by MSCs may minimize damage to the blood–brain barrier by regulating the expression of inflammatory factors in an ICH model (Tang et al., 2021). MSC exosomes containing TSG6 accelerate peripheral nerve regeneration by promoting MMΦ polarization to the M2 type (Li et al., 2022). In our study, we found that treatment with rhTSG6 accelerated hematoma absorption in a mouse model of ICH. RBCs damaged by ICH release neurotoxins that can lead to brain damage and edema, while RBC phagocytosis by MMΦ reduces neurotoxin production, thereby limiting subsequent brain damage (Xia et al., 2022). We found that rhTSG6 treatment reduced hematoma volume and iron deposition in mice subjected to ICH, and that TSG6 can enhance MMΦ polarization from the M1 type to the M2 type and upregulate expression of the scavenger receptor CD36 to enhance phagocytosis, as illustrated by both *in vitro* and *in vivo* experiments. CD36, a member of the class B scavenger receptor family, is crucial for microglia-mediated hematoma clearance (Han et al., 2023). Two *in vivo* studies have reported that targeting CD36 expression promotes hematoma absorption after ICH (Zhao et al., 2020; Li et al., 2024).

pSTAT6 inhibits inflammation and promotes phagocytosis (Cai et al., 2019; Zhang et al., 2019), and silencing the STAT6 gene leads to increased cell loss in brain tissue and exacerbation of long-term functional defects (Cai et al., 2019). IL-4 and IL-13, key STAT6 activators, promote M2-type MMΦ polarization *in vitro* and *in vivo* (He et al., 2020; Xu et al., 2020b). By regulating MMΦ function, IL-4 activates STAT6, which in turn promotes hematoma absorption and neurological function recovery in an ICH model (Xu et al., 2020b). Our results are consistent with this, in that TSG6, similar to IL-4, activates STAT6 following ICH and promotes hematoma clearance and behavioral recovery in mice. However, the TSG6 receptor is unknown. Our results indicate that MMΦ polarization in mice subjected to ICH is induced by TSG6-mediated STAT6 activation.

Our findings suggest that GAS6 is a key downstream target of TSG6/STAT6 signaling. GAS6 expression levels exhibited dynamic changes, reaching a peak on the 3rd day after ICH. Compared with the timing of peak TSG6 and pSTAT6 expression, which occurred on the first day after ICH, the increase in GAS6 expression was relatively delayed. This delayed effect has also been observed for components of the IL-4/STAT6 pathway (Czimmerer et al., 2018; Daniel et al., 2018). This indicates that, although STAT6 is rapidly activated, it can induce second messengers to execute alternative transcription programs and cause stable transcription changes (Liao and Hargreaves, 2020). One study reported that the TSG6/STAT6/GAS6 signaling pathway is vital to alveolar macrophage–mediated phagocytosis of apoptotic cells (Nepal et al., 2019). We observed that TSG6 increased STAT6 phosphorylation, thereby promoting GAS6 expression, and that treatment with a STAT6 phosphorylation inhibitor prevented this increase in GAS6 expression. This finding indicates that STAT6 is an upstream signaling molecule that upregulates GAS6 expression through the TSG6/STAT6 signaling pathway. Increased GAS6 expression activated the GAS6/TAM system (including AXL, MERTK, and TYRO3). A previous study showed that mutations in the GAS6 gene decreased total serum AXL protein and mRNA levels, and that this was positively correlated with an elevated risk of bleeding after intravenous thrombolysis, in patients with ischemic stroke (Guo et al., 2021). In mice subjected to ICH, the absence of AXL and MERTK resulted in reduced phagocytosis of RBCs, slowed hematoma clearance, delayed recovery of neural function, increased iron deposition, and reduced selective activation of macrophages after ICH (Chang et al., 2018). We found that the TSG6/STAT6 signaling pathway increased GAS6/TAM system expression. The GAS6/TAM system inhibits microglial neuroinflammatory activity in addition to preserving their phagocytic capacity (Ji et al., 2013). Our study showed that GAS6/TAM system expression is increased when the TSG6/STAT6 signaling axis is activated and decreased when it is inhibited. This result suggests that the GAS6/TAM system plays an essential role in mediating at least some of the therapeutic effects of TSG6/STAT6 on ICH in a mouse model. While this study focused on TSG6 interactions with the STAT6/GAS6 pathway, it is also important to consider other inflammatory mediators that could work synergistically with TSG6 in the context of hematoma resolution. Previous research has demonstrated that IL-10 and peroxisome proliferator-activated receptor gamma also drive microglial polarization toward the M2 phenotype and enhance phagocytosis (Cianciulli et al., 2015; Song et al., 2017). These molecules can interact with TSG6, forming a complex network of signaling events that regulate the immune response and facilitate resolution of hemorrhagic lesions (Fu et al., 2022). Therefore, exploring the interactions between TSG6 and other inflammatory modulators could provide further insight into its role in hematoma clearance and lead to the identification of additional therapeutic targets.

Although our findings suggest that TSG6 promotes STAT6 phosphorylation, it is unclear whether TSG6 exerts this effect directly or indirectly through mediators. For example, exogenous rhTSG6 may activate STAT6 by binding to the CD44 receptor (Day and Milner, 2019; Yang et al., 2019) or directly enter the cell through endocytosis to bind to STAT6. Similarly, it remains unclear whether the intracellular TSG6 acts directly on STAT6 or in an autocrine through its extracellular domain by binding to a receptor, thereby activating STAT6. Therefore, further exploration is needed to identify interaction sites or mediators between TSG6 and STAT6. Additionally, randomized, double-blind, placebo-controlled clinical trials need to be performed to confirm the safety and efficacy of TGS6 as a treatment for promoting hematoma absorption and improving prognosis in patients with ICH.

This study identified endogenous TSG6 as a key molecule inducing MMΦ polarization and phagocytosis to promote hematoma absorption and neurological recovery after ICH, both *in vitro* and *in vivo*. STAT6, a crucial downstream signaling molecule of TSG6, mediated its protective effects by promoting GAS6 expression. Thus, the TSG6/STAT6/GAS6 signaling pathway regulates MMΦ activation, promotes hematoma absorption, reduces brain damage, and accelerates restoration of neural function after ICH, and is therefore a potential therapeutic target. rhTSG6 is expected to become a new drug for ICH treatment that should be investigated further to promote its clinical development.

## Additional files:

***Additional Figure 1:*** *Experimental flow chart.*

Additional Figure 1 Experimental flow chart.
(A) TSG6 expression in patients with ICH and a mouse model of ICH. (i) TSG6 expression in patients with ICH from the GEO database; (ii) TSG6 expression in mice subjected to ICH, as detected by WB, RT-PCR, and IF. (iii) CCK-8 assay of BV2 cells treated with different concentrations of hemin to simulate ICH in vitro. (iv) TSG6 expression in an in vitro model of ICH, as detected by WB, RT-PCR, and IF. (B) The effect of TSG6 on hematoma resolution, neuronal damage, and neurobehavioral recovery in a mouse model of ICH. (i) Effect of TSG6 administration on hematoma resolution, as detected by hematoma volume, hemoglobin content, and WB. (ii) Effect of TSG6 knockdown on neuronal damage and neurobehavior, as detected by HE staining, iron staining, Nissl staining, and neurobehavioral tests. (iii) Effect of TSG6 administration on neuronal damage and neurobehavior, as detected by HE staining, iron staining, TUNEL, Nissl staining, and neurobehavioral tests. (C) The effect of TSG6 on MMΦ polarization and phagocytic function post-ICH. (i) The effect of TSG6 on MMΦ polarization post-ICH *in vivo* and *in vitro*, as detected by WB and IF. (ii) The effect of TSG6 on MMΦ phagocytic function post-ICH, as detected by WB and an in vitro phagocytosis test. (D) Changes in downstream components of the TSG6 signaling pathway. (i) Dynamic changes in pSTAT6/STAT6 expression levels in mice subjected to ICH, as detected by WB. (ii) The effect of TSG6 administration and TSG6 knockdown on pSTAT6/STAT6 expression in mice subjected to ICH, as detected by WB. (iii) The effect of TSG6 after inhibiting STAT6 phosphorylation with AS1517499, as detected by WB, hematoma volume, iron staining, HE staining, TUNEL, and Nissl staining. (iv) Analysis of possible downstream targets of TSG6/STAT6, as detected by WB. CCK-8: Cell Counting Kit-8; DMSO: dimethyl sulfoxide; HE: hematoxylin-eosin staining; ICH; intracerebral hemorrhage; IF: immunofluorescence; MMΦ: microglia/macrophages; PBS: phosphate-buffered saline; RT-PCR: real-time polymerase chain reaction; STAT6: signal transducer and activator of transcription 6; TSG6: tumor necrosis factor-α-stimulated gene 6; TUNEL: transferase-mediated dUTP nick-end labeling; WB: western blot.

***Additional Figure 2:*** *Hematoma volumes at different time points in mice subjected to ICH.*

Additional Figure 2 Hematoma volumes at different time points in mice subjected to ICH.
(A, B) Hematomas appeared in the basal ganglia area of mice 6 hours after collagenase injection, with the largest hematoma volume seen at 24 hours and then gradually decreasing. At 7 days after collagenase injection, there was no significant difference in hematoma volume compared with mice from the sham surgery group. All data are reported as mean ± SD. ^*^P < 0.05, ^****^P < 0.0001, vs. sham group. Two-way analysis of variance followed by Tukey’s *post hoc* test was used in B. ICH: Intracerebral hemorrhage.

***Additional Figure 3:*** *Effects of TSG6 on brain hematoma at 3 days after ICH in mice.*

Additional Figure 3 Effects of TSG6 on brain hematoma at 3 days after ICH in mice.
(A-D) HE staining showed more MMΦ around the hematoma in the ICH + rhTSG6 group than in the ICH + PBS group (A, B) and more MMΦ around the hematoma in the ICH + sh-ctrl groups than in the ICH + sh-TSG6 group (C, D). All data are reported as mean ± SD. ^*^P < 0.05, ^***^P < 0.001. Two-tailed Student’s *t*-test was used in B and D. HE: Hematoxylin-eosin staining; ICH: intracerebral hemorrhage; MMΦ: microglia/macrophages; PBS: phosphate-buffered saline; TSG6: tumor necrosis factor-α-stimulated gene 6.

***Additional file 1:*** *Experimental details.*

Additional file 1 Experimental details

## Data Availability

*All data relevant to the study are included in the article or uploaded as Additional files.*
