# Supplementary material for: Tumor necrosis factor-α–stimulated gene 6 promotes hematoma clearance after intracerebral hemorrhage in a mouse model
Source: Neural Regen Res. 2025 Jun 19;21(8):3629–40. doi: 10.4103/NRR.NRR-D-24-00968 (PMC13452657; doi:10.4103/NRR.NRR-D-24-00968)
Supplement: Supplementary file 1 [file NRR-21-3629_Suppl1.pdf]

Additional file 1: Experimental details

Tissue preparation for western blot and real-time polymerase chain reaction

Mice were anesthetized with an intraperitoneal injection of pentobarbital sodium and then perfused with phosphate buffered saline. After the whole brain was removed, the brain tissues around the hematoma were further separated and placed in a cryopreservation tube, and then the samples were instant used or transferred to liquid nitrogen for storage for further experiments.

Staining of brain tissue sections

Mice were anesthetized with an intraperitoneal injection of pentobarbital sodium 3 days after intracerebral hemorrhage (ICH) induction and then perfused with phosphate buffered saline and 4% paraformaldehyde. The brain tissue was fixed with 4% paraformaldehyde overnight at 4℃, followed by immersion in 20% and 30% sucrose. The tissue was embedded in optimal cutting temperature compound gel, and 5-μm-thick coronal slices were cut from the vicinity of the pinhole with a cryostat (RM2016, Leica Instrument Co., Ltd, Shanghai, China). These brain slices were stored at 4℃ for the following experiments.

Transferase-mediated dUTP nick-end labeling staining

Following the manufacturer's instructions, we conducted a transferase-mediated dUTP nick-end labeling (TUNEL) staining test on brain slices from each group. Utilizing a Nikon eclipse C1 microscope (Tokyo, Japan), we observed the brain slices, focusing on the area near the hematoma. Image J software was then used for analysis.

Nissl staining

The brain sections were dewaxed and rehydrated before being placed in the toluidine blue solution at a temperature between 55℃ and 60℃ for 1 hour of dyeing. Afterward, they were rinsed with single distilled water and then gradually dehydrated in a series of concentration gradient ethanol solutions (70%, 80%, 95%, and 100%). Finally, the slices were immersed in 100% xylene and the cover glass was sealed with neutral balsam. To count the number of apoptotic and viable cells in the area around the hematoma, an observer who was unaware of the experimental design used a laser scanning confocal microscope (Zeiss LSM800, München, Germany) and a microscope (Olympius BX60, Tokyo, Japan) to observe the pictures.

Immunofluorescent analysis

Cell immunofluorescence

After harvesting, cells were fixed with paraformaldehyde, we applied double labeling of two primary antibodies and kept them in a 4℃ environment overnight. Afterward, we incubated the sections with the corresponding secondary antibodies, and counterstained them with 4′,6-diamidino-2-phenylindole (DAPI). Finally, we employed a laser scanning confocal microscope for analysis.

Tissue immunofluorescence

After dewaxing and antigen retrieval, the sections were incubated overnight at 4 °C with a combination of two primary antibodies for double labeling. The sections were then incubated with the corresponding secondary antibodies and counterstained with DAPI. Finally, images were acquired using a Zeiss LSM800 laser scanning confocal microscope (München, Germany).

Iron staining

We used the Prussian blue iron stain kit (with eosin or enhance with diaminobenzidine (Solarbio Science&Technology Co., Ltd., Beijing, China) to detect iron deposition in brain tissue sections. After washing the sections with PBS and H2O2, they were incubated with freshly prepared Perls solution (5% potassium ferrocyanide [Sigma Aldrich]/10% hydrochloric acid) for 1 hour, followed by three washes with PBS. Subsequently, diaminobenzidine or Eosin dye was applied for 3 minutes, and the images were viewed under microscopes (Olympus BX60, Tokyo, Japan and Zeiss LSM800).

Neurobehavioral tests

Neurobehavioral tests were conducted on all mice to assess any abnormalities in the nervous system prior to the experiment, as well as 1 day before, and 1, 3, 5, 7, and 14 days after ICH.

Garcia score

Investigators, blind to the experimental treatment protocol, assessed several indices, such as symmetrical limb movement, spontaneous activity, climbing, front paw extension, reaction to tentacles, and body proprioception. Each test was graded on a scale of 0 (worst) to 3 (best), and the nervous system score of the mice was calculated by summing the scores (Xu et al., 2020).

Corner turn

In the angle test, mice were placed in front of a 30° angle constructed from two pieces of cardboard measuring 30 and 20 cm, respectively. The mice could turn left or right to exit the corner, and when one side of the body's nerve function was impaired, the mice were more likely to turn to the other side. Following ten trials per mouse, the proportion of mice turning right was recorded (Zhang et al., 2002).

Adaptive removal test

To evaluate the asymmetry of sensorimotor and tactile responses, 2 mm × 3 mm adhesive tape was applied to the left paw (damaged side, opposite side of brain injury). The tactile response was recorded by noting the time when the injured front paw was initially contacted with the mouth and when the tape was taken off from the damaged front paw with the mouth. The observation time was limited to 100 seconds (Hoda et al., 2014).

Cylinder test

The use of the front paws of mice was evaluated to assess asymmetry. Mice were placed in a cylindrical glass bottle of 10 cm diameter and 20 cm height and recorded from the top for 5 minutes. The videotape was analyzed in slow motion, and the use of the front paws (left, right, or both) was

recorded at the first contact with the cylinder wall and during lateral exploration. Uninjured mice generally did not show a preference for any of their front paws, whereas injured mice exhibited reduced use of the left paw (injured side or injured opposite side) depending on the severity of the injury. The formula for calculating the asymmetry of the front paw use was: (right - left)/(left + right) × 100% (Yu et al., 2019).

Foot fault test

This study evaluated sensorimotor coordination during spontaneous. A mouse was placed on a wire mesh surface (50 cm [L] × 25 cm [W] × 10 cm [H]) with an opening of 2.25 cm^2 (1.5 cm × 1.5 cm), and the movements were recorded for 1 minute from below the grid. An observer unaware of the groupings analyzed the total steps of the videotape and the number of foot failures in the left limb (the side opposite to the lesion). When the mouse misplaced the damaged front or rear paw, causing the paw to cross the mesh, it was considered a foot failure. The data were expressed as the percentage of foot failure in the total steps of the claw (Modo et al., 2000).

Antibodies

The following antibodies were used for western blot analysis including the primary antibodies: rabbit anti-TSG6 (1:1000, GeneTex, Inc., Irvine, CA, USA, Cat# GTX64605, RRID: AB_3668746), rabbit anti-GAS6 (1:1000, GeneTex, Inc., Cat# GTX134747, RRID:AB_2887339), rabbit anti-IL6 (1:500, Abcam, Cambridge, UK, Cat# ab290735, RRID:AB_3064891), rabbit anti-CD16/32 (1:1000, Abcam, Cat# ab228971, RRID: AB_3668747), rabbit anti-iNOS (1:1000, Cell Signaling Technology, Beverly, MA, USA, Cat# 13120, RRID:AB_2687529), mouse anti-Arg1 (1:500, Servicebio Biotechnology Co., Ltd, Wuhan, China, Cat# 66129-1-Ig, RRID:AB_2881528), rabbit anti-CD163 (1:500, Cell Signaling Technology, Cat# 93498, RRID:AB_2800204), rabbit anti-CD206 (1:2000, Cell Signaling Technology, Cat# 24595, RRID:AB_2892682), rabbit anti-CD36 (1:2000, Abcam, Cat# ab133625, RRID:AB_2716564), rabbit anti-STAT6 (1:1000, Cell Signaling Technology, Cat# 5397, RRID:AB_11220421), rabbit anti-pSTAT6 (1:500, Cell Signaling Technology, Cat# 56554, RRID:AB_2799514), rabbit anti-AXL (1:1000, Abcam, Cat# ab215205, RRID:AB_2924328), rabbit anti-MERTK (1:1000, Abcam, Cat# ab300136, RRID:AB_2936858), rabbit anti-Tubulin (1:2000, Sanying Biotechnology Co., Ltd, Wuhan, China, Cat# 11224-1-AP, RRID:AB_2210206), rabbit anti-GAPDH (1:4000, Sanying Biotechnology Co., Ltd, Cat# 10494-1-AP, RRID: AB_2263076), and the secondary antibodies: Anti-rabbit IgG (H+L)(1:12000, Cell Signaling Technology, Cat# 5366, RRID: AB_10693812), Anti-mouse IgG (H+L)(1:10000, Cell Signaling Technology, Cat# 5470, RRID:AB_10696895).

The following antibodies were used for immunofluorescent analysis including the primary antibodies: mouse anti-GFAP (1:200, Wuhan Servicebio Biotechnology Co., Ltd, Cat# GB12090, RRID: AB_3668748), mouse anti-IBA1 (1:200, Wuhan Servicebio Biotechnology Co., Ltd, Cat# GB12105, RRID: AB_2922434), rabbit anti-IBA1 (1:500, FUJIFILM Wako Pure Chemical Corporation, Osaka, Japan, Cat# 019-19741, RRID: AB_839504), rabbit anti-TSG6 (1:100, GeneTex, Inc., Cat# GTX64605, RRID: AB_3668746), rabbit anti-CD16/32 (1:200, Abcam, Cat# ab228971, RRID: AB_3668747), mouse anti-Arg1 (1:100, Wuhan Servicebio Biotechnology Co., Ltd, Cat# 66129-1-Ig, RRID:

AB_2881528), mouse anti-NEUN (1:200, Sanying Biotechnology Co., Ltd, Cat# 66836-1-Ig, RRID: AB_2882179), and the secondary antibodies: 594-Goat Anti-Rabbit IgG(H+L) (1:200, Sanying Biotechnology Co., Ltd, Cat# SA00013-4, RRID:AB_2810984), 488 Goat Anti-Rabbit IgG (1:200, Sanying Biotechnology Co., Ltd, Cat# SA00013-2, RRID: AB_2797132), 594 Goat Anti-Mouse IgG (1:200, Sanying Biotechnology Co., Ltd, Cat# SA00013-3, RRID: AB_2797133).

References

Hoda MN, Bhatia K, Hafez SS, Johnson MH, Siddiqui S, Ergul A, Zaidi SK, Fagan SC, Hess DC (2014) Remote ischemic perconditioning is effective after embolic stroke in ovariectomized female mice. Transl Stroke Res 5:484-490.

Modo M, Stroemer RP, Tang E, Veizovic T, Sowniski P, Hodges H (2000) Neurological sequelae and long-term behavioural assessment of rats with transient middle cerebral artery occlusion. J Neurosci Methods 104:99-109.

Xu J, Chen Z, Yu F, Liu H, Ma C, Xie D, Hu X, Leak RK, Chou SHY, Stetler RA, Shi Y, Chen J, Bennett MVL, Chen G (2020) IL-4/STAT6 signaling facilitates innate hematoma resolution and neurological recovery after hemorrhagic stroke in mice. Proc Natl Acad Sci U S A 117:32679-32690.

Yu J, Moon J, Jang J, Choi JI, Jung J, Hwang S, Kim M (2019) Reliability of behavioral tests in the middle cerebral artery occlusion model of rat. Lab Anim 53:478-490.

Zhang L, Schallert T, Zhang ZG, Jiang Q, Arniego P, Li Q, Lu M, Chopp M (2002) A test for detecting long-term sensorimotor dysfunction in the mouse after focal cerebral ischemia. J Neurosci Methods 117:207-214.
